# Validation of a survival-risk score (SRS) in relapsed/refractory CLL patients treated with idelalisib–rituximab

**DOI:** 10.1038/s41408-020-00358-3

**Published:** 2020-09-16

**Authors:** Massimo Gentile, Enrica Antonia Martino, Andrea Visentin, Marta Coscia, Gianluigi Reda, Paolo Sportoletti, Francesca Romana Mauro, Luca Laurenti, Marzia Varettoni, Roberta Murru, Annalisa Chiarenza, Ernesto Vigna, Francesco Mendicino, Eugenio Lucia, Sabrina Bossio, Anna Grazia Recchia, Riccardo Moia, Daniela Pietrasanta, Giacomo Loseto, Ugo Consoli, Ilaria Scortechini, Francesca Maria Rossi, Antonella Zucchetto, Hamdi Al-Janazreh, Candida Vitale, Giovanni Tripepi, Graziella D’Arrigo, Ilaria Angeletti, Riccardo Bomben, Antonino Neri, Giovanna Cutrona, Gilberto Fronza, Francesco Di Raimondo, Gianluca Gaidano, Antonio Cuneo, Robin Foà, Manlio Ferrarini, Livio Trentin, Valter Gattei, Fortunato Morabito

**Affiliations:** 1 Hematology Unit AO of Cosenza, Cosenza, Italy; 2 Biothecnology Research Unit, AO of Cosenza, Cosenza, Italy; 3 Department of Medicine, Hematology and Clinical Immunology Branch, University of Padova, Padova, Italy; 4 Division of Hematology, A.O.U. Città della Salute e della Scienza di Torino, Torino, Italy; 5 Ematologia, Fondazione IRCCS Ca’ Granda Ospedale Maggiore Policlinico di Milano, Milano, Italy; 6 Centro di Ricerca Emato-Oncologica (CREO), University of Perugia, Perugia, Italy; 7 Hematology, Department of Translational and Precision Medicine, ‘Sapienza’ University, Rome, Italy; 8 Fondazione Universitaria Policlinico A Gemelli di Roma, Roma, Italy; 9 Division of Haematology, Fondazione IRCCS Policlinico San Matteo, Pavia, Italy; 10 Hematology and Stem Cell Transplantation Unit, Ospedale A. Businco, Cagliari, Italy; 11 Division of Hematology, Policlinico, Department of Surgery and Medical Specialties, University of Catania, Catania, Italy; 12 Division of Hematology, Department of Translational Medicine, University of Eastern Piedmont, Novara, Italy; 13 Division of Hematology, Azienda Ospedaliera SS Arrigo e Biagio e Cesare Arrigo, Alessandria, Italy; 14 Hematology and Cell Therapy Unit, IRCCS-Istituto Tumori ‘Giovanni Paolo II’, Bari, Italy; 15 Hematology Department, G. Garibaldi Hospital, Catania, Italy; 16 Clinica di Ematologia Ospedali Riuniti, Ancona, Italy; 17 Clinical and Experimental Onco-Hematology Unit, Centro di Riferimento Oncologico di Aviano (CRO) IRCCS, Aviano (PN), Italy; 18 Hematology and Bone Marrow Transplant Unit, Hemato-Oncology Department, Augusta Victoria Hospital, East Jerusalem, Israel; 19 CNR-IFC, Research Unit of Reggio Calabria, Reggio Calabria, Italy; 20 Reparto di Oncoematologia Azienda Ospedaliera Santa Maria di Terni, Terni, Italy; 21 Molecular Pathology Unit, IRCCS Ospedale Policlinico San Martino, Genova, Italy; 22 Mutagenesis and Cancer Prevention Unit, IRCCS Ospedale Policlinico San Martino, Genoa, Italy; 23 Hematology Section, Department of Medical Sciences, University of Ferrara, Ferrara, Italy; 24 Department of Experimental Medicine, University of Genoa, Genoa, Italy

**Keywords:** Chronic lymphocytic leukaemia, B-cell lymphoma

Dear Editor,

The identification of prognostic models for overall survival (OS) of relapsed/refractory (R/R) chronic lymphocytic leukemia (CLL) treated with novel target drugs, such as B-cell receptor (BCR) and BCL-2 inhibitors, represents an unmet clinical need. Recently, our group proposed a survival-risk score for real-life R/R CLL patients treated with Ibrutinib (SRS_I_).^1^ This SRS_I_ is based on three laboratory parameters, β2 microglubulin (β2 M, 1 point for cases with β2 M > 5 mg/L), lactic dehydrogenase values (LDH, 2 points for cases with LDH > upper limit of normal), and hemoglobin level (2 points for men with hemoglobin < 11 g/L and 2 points for women with hemoglobin < 12 g/L) (Supplementary Table 1), and represents a powerful and easily applicable prognostic tool for the prediction of OS. Indeed, unique information originated from a real-life retrospective study with a huge number of R/R CLL patients treated either with chemoimmunotherapy or with new drugs, and proposed a comprehensive risk score for the OS prediction. On the other hand, the randomized trial comparing Idela-R versus R showed the greater performance of the Idela-R in all experimental settings [i.e., IGHV-unmutated cases, del(17p) cases] (2). Moreover, the final results of the same randomized trial (3) reported that the presence of del(17p) or *TP53* mutations did not negatively affect clinical outcomes among patients treated with Idela/R. The present retrospective, multicenter study was undertaken with the aim of testing whether SRS_I_ was also useful for R/R CLL patients treated with idelalisib–rituximab (Idela-R), thus further refining the role of some prognostic factors in predicting OS in a setting homogeneously treated for patients.

Overall, 142 CLL patients present in the CLL databases from 15 Italian centers (see Supplementary Appendix for details), could be included in this analysis. The majority of patients were Binet stages B and C (94.6%). The median age was 75.1 years (range: 37.1–91) and 98 cases (69%) were male. The median number of previous therapies was 3 (range: 1–9). Fifty-six patients discontinued Idela-R due to toxicity, 20 for CLL progression, and 6 for Richter transformation; 2 responding cases underwent an allogeneic stem cell transplantation. The baseline patients’ features are listed in Supplementary Table 2. After a median follow-up of 1.6 years, 45 patients had died.

The relationship between the SRS_I_ parameters and OS was assessed. All three SRS_I_ parameters were associated with OS in univariate analysis and in a multiple Cox regression analysis (Table 1). Thirty-six patients were classified at low risk, 76 at intermediate risk and 30 at high risk according to the SRS_I_. The OS of the three patient groups was significantly different (Fig. 1), and an overlap among curves was not observed across time. Low-risk patients had a 2-year OS probability of 88.6% (HR = 1, reference category), intermediate-risk patients of 69.6% (HR = 3.5, 95% CI: 1.2–10.2, *P* = 0.022), and high-risk patients of 54.3% (HR = 8.0, 95% CI: 2.7–23.7, *P* < 0.0001) (Fig. 1). The *C* statistic was 0.66 (*P* < 0.001) for OS prediction (Fig. 1), a figure reasonably close to the well-known critical cutoff of 0.7 useful to counsel an individual patient. Of note, no statistically significant differences in terms of the number of lines of therapy or of discontinuation of idelalisib for toxicity were observed in the three risk categories.

**Table 1:** Univariate and multivariate analyses.

Features	Univariate analysis		Multivariate analysis (SRS_I_ model)		Multivariate analysis (BALL model)		Multivariate analysis (SRS_I_ + biological parameter model)	
	HR (95% CI)	*P*	HR (95% CI)	*P*	HR (95% CI)	*P*	HR (95% CI)	*P*
Hemoglobin <110 g/L for women <120 g/L for men	3.63 (1.87–7.05)	<0.0001	2.36 (1.15–4.84)	0.02	2.19 (1.05–4.58)	0.036	2.37 (1.15–4.89)	0.019
β2 M ≥ 5 mg/L	3.98 (2.15–7.36)	<0.0001	2.25 (1.13–4.48)	0.021	2.23 (1.12–4.46)	0.023	2.23 (1.12–4.46)	0.022
LDH > ULN	3.18 (1.73–5.87)	<0.0001	2.29 (1.21–4.33)	0.011	2.21 (1.16–4.19)	0.016	2.03 (1.05–3.94)	0.035
Time from the last therapy <24 months	2.37 (1.01–5.6)	0.049	–	–	1.51 (0.62–3.67)	0.365	–	–
Age >65 years	0.66 (0.32–1.38)	0.274	–	–	–	–	–	–
17p deletion positive	2.07 (1.14–3.78)	0.017	–	–	–	–	1.59 (0.85–2.96)	0.15
IGHV unmutated	1.3 (0.93–1.74)	0.13	–	–	–	–	–	–

*HR* hazard ratio, *95% CI* 95% confidence interval, *β2 M* β2 microglobulin, *ULN* upper limit of normal.

**Fig. 1:**
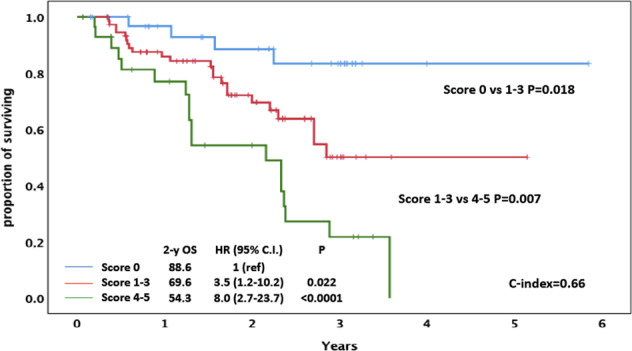
Overall survival of the entire population of 142 CLL patients according to SRS_I_. (β2 M ≤ 5 = 0 points; β2 M > 5 = 1 point; hemoglobin > 11 g/L for women and > 12 g/L for men = 0 points; hemoglobin ≤ 11 g/L for women and ≤ 12 g/L for men = 2 points; LDH ≤ UNL = 0 points; LDH > UNL = 2 points; total score 0 = low risk; score 1–3 = intermediate risk; score 4–5 = high risk).

Recently, a retrospective pooled cohort study based on an international collaboration collected information from ~2500 R/R CLL patients treated either with chemoimmunotherapy or with new drugs (Ibrutinib, Idelalisib, or Venetoclax), and proposed a comprehensive risk score for the OS prediction, based on four widely accessible parameters: β2 M, anemia, LDH, and time from last therapy, a.k.a. BALL score (Supplementary Table 2).^2^ According to the BALL score, 46 patients of the present study were classified at low risk, 77 at intermediate risk and 19 at high risk. Although significant differences in OS were found between low-risk versus intermediate-risk patients (*P* < 0.001), this stratification failed to detect significant differences between intermediate-risk versus high-risk cases (*P* = 0.057) (Supplementary Fig. 1). Since the BALL score differs from SRS_I_ for the presence of the time from last therapy (≥24 versus <24 months) variable, the prognostic power of this parameter was also evaluated in our cohort. At univariate analysis, time from last therapy was significantly associated with OS (HR = 2.27; 95% CI: 1.01–5.6; *P* = 0.049) (Table 1), but it lost its prognostic significance when forced into a multivariate model, together with the three parameters of the SRS_I_ score, which remained independently associated with OS (Table 1).

The above differences were somewhat expected, given that the BALL score was designed for R/R CLL patients undergoing salvage treatment, using either biological agents or chemoimmunotherapy, whereas the SRS_I_ score was specifically adapted for R/R patients undergoing BCR-inhibitor treatment. Probably, the use of new drugs as salvage therapy in this setting of patients can overcome the negative prognostic impact of a short time from last therapy (<24 months), as previously reported by our group in the ibrutinib setting.^1^

Finally, we evaluated the prognostic significance of two biological parameters (i.e., IGHV mutational status and 17p deletion) in our series. At univariate analysis, 17p deletion (HR: 2.07; 95% CI: 1.14–3.78; *P* = 0.017) and not the IGHV status (HR: 1.3; 95% CI: 0.93–1.74; *P* = 0.13) remained significantly associated with survival (Table 1). Nonetheless, 17p deletion failed to maintain its independent prognostic power when added to LDH, β2-M values, and hemoglobin levels in a multivariate model, while all three SRS_I_ parameters remained independently associated with survival (Table 1). These data are in line with the exploratory analysis performed in the Study 116 trial,^3,4^ indicating that the presence of 17p deletion does not negatively affect the survival of R/R CLL patients who are generally ineligible for standard chemotherapy, but can be treated with Idela-R.^3,4^ At univariate analysis, age (HR 0.66; 95% CI: 0.32–1.38; *P* = 0.274) did not remain significantly associated with survival (Table 1).

Overall, the present data indicate that parameters related to tumor burden (i.e., LDH and β2-M values) and to bone marrow reserve (i.e., hemoglobin level) represent the most important prognostic markers of survival in R/R CLL patients receiving Idela-R. This is similar to what we found for Ibrutinib, and suggests that these criteria may be universally valuable for BCR inhibitors [survival risk score for real-life R/R CLL patients treated with Ibrutinib or with Idela-R (SRS_II_)]. Furthermore, SRS_II_ is a simple and parsimonious prognostic score, which is unlikely to be affected by missing genetic data, when employed in clinical practice. Nevertheless, in the absence of ad hoc phase III randomized studies, the final choice of the most appropriate BCR-inhibitor therapy is frequently dictated by the presence of comorbidities or by the expected toxicity drug profiles. In this context, the proposed SRS_II_ may represent an additional and easily applicable tool for the prediction of OS in R/R CLL patients treated with BCR inhibitors. However, in the current era of CLL treatments that have relegated therapy with Idela-R to the third or potentially fourth line of treatment, patients with high-risk SRS score should be considered for a combination of new drugs, aimed at attaining undetectable minimal residual disease and treatment-free remissions.

## Supplementary information

Supplementary Materials

Supplementary Figure 1
