# Supplementary material for: Validation of a survival-risk score (SRS) in relapsed/refractory CLL patients treated with idelalisib–rituximab
Source: Blood Cancer J. 2020 Sep 16;10(9):92. doi: 10.1038/s41408-020-00358-3 (PMC7494850; doi:10.1038/s41408-020-00358-3)
Supplement: Supplementary file 1 — Supplementary Materials [file 41408_2020_358_MOESM1_ESM.docx]

**SUPPLEMENTARY APPENDIX CONTENTS**

**VALIDATION OF A SURVIVAL-RISK SCORE (SRS) IN RELAPSED/REFRACTORY CLL PATIENTS TREATED WITH RITUXIMAB-IDELALISIB**

Massimo Gentile^1,2^, Enrica Antonia Martino^1^, Andrea Visentin^3^, Marta Coscia^4^, Gianluigi Reda^5^, Paolo Sportoletti^6^, Francesca Romana Mauro^7^, Luca Laurenti^8^, Marzia Varettoni^9^, Roberta Murru^10^, Annalisa Chiarenza^11^, Ernesto Vigna^1,2^, Francesco Mendicino^1^, Eugenio Lucia^1^, Sabrina Bossio^2^, Anna Grazia Recchia^2^, Riccardo Moia^12^, Daniela Pietrasanta^13^, Giacomo Loseto^14^, Ugo Consoli^15^, Ilaria Scortechini^16^, Francesca Maria Rossi^17^, Antonella Zucchetto^17^, Hamdi Al-Janazreh^18^, Candida Vitale^4^, Giovanni Tripepi^19^, Graziella D’Arrigo^19^, Ilaria Angeletti^20^, Riccardo Bomben^17^, Antonino Neri^5^, Giovanna Cutrona^21^, Francesco Di Raimondo^11^, Gianluca Gaidano^12^, Antonio Cuneo^22^, Robin Foà^7^, Manlio Ferrarini^23^, Livio Trentin^3^, Valter Gattei^17^, Fortunato Morabito^2,18^.

^1^Hematology Unit AO of Cosenza, Cosenza, Italy; ^2^Biothecnology Research Unit, AO of Cosenza, Cosenza, Italy; ^3^Department of Medicine, Hematology and Clinical Immunology Branch, University of Padova, Padova, Italy; ^4^Division of Hematology, A.O.U. Città della Salute e della Scienza di Torino, Torino, Italy; ^5^Ematologia, Fondazione IRCCS Ca'Granda Ospedale Maggiore Policlinico di Milano, Italy; ^6^Centro di Ricerca Emato-Oncologica (CREO), University of Perugia, Perugia, Italy; ^7^Hematology, Department of Translational and Precision Medicine, 'Sapienza' University, Rome, Italy; ^8^Fondazione Universitaria Policlinico A Gemelli di Roma, Roma, Italy; ^9^Division of Haematology, Fondazione IRCCS Policlinico San Matteo, Pavia, Italy; ^10^Hematology and Stem Cell Transplantation Unit, Ospedale A. Businco, Cagliari, Italy; ^11^Division of Hematology, Policlinico, Department of Surgery and Medical Specialties, University of Catania, Italy; ^12^Division of Hematology, Department of Translational Medicine, University of Eastern Piedmont, Novara, Italy; ^13^Division of Hematology, Azienda Ospedaliera SS Arrigo e Biagio e Cesare Arrigo, Alessandria, Italy; ^14^Hematology and Cell Therapy Unit, IRCCS-Istituto Tumori 'Giovanni Paolo II', Bari, Italy; ^15^Hematology Department, G. Garibaldi Hospital, Catania, Italy; ^16^Clinica di Ematologia Ospedali Riuniti, Ancona, Italy; ^17^Clinical and Experimental Onco-Hematology Unit, Centro di Riferimento Oncologico di Aviano (CRO) IRCCS, Aviano (PN), Italy; ^18^Hematology and Bone Marrow Transplant Unit, Hemato-Oncology Department, Augusta Victoria Hospital, East Jerusalem, Israel; ^19^CNR-IFC, Research Unit of Reggio Calabria, Reggio Calabria, Italy; ^20^Reparto di Oncoematologia Azienda Ospedaliera Santa Maria di Terni, Terni, Italy; ^21^Molecular Pathology Unit, IRCCS Ospedale Policlinico San Martino, Genova, Italy; ^22^Hematology Section, Department of Medical Sciences, University of Ferrara, Italy; ^23^Department of Experimental Medicine, University of Genoa, Genoa, Italy.

**Supplementary Methods**

**Supplementary References**

**Supplementary Tables**

**Supplementary Figure legends**

**Supplementary Methods**

**Patients**

CLL databases from 15 Italian centers (Hematology Unit AO of Cosenza; Division of Hematology, Cosenza; Hematology, Department of Translational and Precision Medicine, 'Sapienza' University, Rome; Hematology Unit, IRCCS Ca' Granda Policlinico-Università degli Studi di Milano, Milano; Centro di Ricerca Emato-Oncologica (CREO), University of Perugia, Perugia; Fondazione Universitaria Policlinico A Gemelli di Roma, Roma; Division of Hematology, A.O.U. Città della Salute e della Scienza di Torino, Torino; Division of Haematology, Fondazione IRCCS Policlinico San Matteo, Pavia; Hematology and Stem Cell Transplantation Unit, Ospedale A. Businco, Cagliari; Division of Hematology, Policlinico, Department of Surgery and Medical Specialties, University of Catania; Division of Hematology, Department of Translational Medicine, University of Eastern Piedmont, Novara; Division of Hematology, Azienda Ospedaliera SS Arrigo e Biagio e Cesare Arrigo, Alessandria; Hematology and Cell Therapy Unit, IRCCS-Istituto Tumori 'Giovanni Paolo II', Bari; Hematology Department, G. Garibaldi Hospital, Catania; Clinica di Ematologia Ospedali Riuniti, Ancona; Reparto di Oncoematologia Azienda Ospedaliera Santa Maria di Terni, Terni; Department of Medicine, Hematology and Clinical Immunology Branch, University of Padova, Padova) were set up for research purposes and analysed in the context of a institutional Italian multicenter study group on CLL (Campus CLL). The databases contained clinical information such as age, sex, date of diagnosis, Rai and Binet stage, laboratory parameters, biological markers, treatment history, date of last follow-up or death, which were abstracted from clinical records at the time of inclusion and updated on an ongoing basis. The 15 databases included 142 consecutive cases of R/R CLL treated with idelalisib plus rituximab between June 2013 and May 2019. All 142 cases were evaluable for SRS_I_ score since information regarding β2-M level, haemoglobin and LDH values were fully available.

**Immunoglobulin gene mutation, cytogenetic and FISH analyses**

*IGHV* mutation analysis and FISH were performed at the reference laboratory of each participating center. The *IGHV* mutation status was tested on tumor DNA collected at diagnosis, and was assessed according to the ERIC guidelines.^1^ Sequences that differed by more than 2% from their corresponding germ-line sequence were considered as mutated.^1-3^ FISH analysis was performed on nuclei extracted from fresh or frozen peripheral blood mononuclear cells collected at diagnosis. The probe used for 17p deletion analysis was LSIp53 (Abbott). At least 200 interphase cells were examined. The presence of 17p deletion abnormality was scored when the percentage of nuclei with the abnormality was above each laboratory’s internal cut-off defined as the mean plus 3 standard deviations of the frequency of normal control cells exhibiting the abnormality.^4^ The complex karyotype was defined by the presence of at least 3 chromosome aberrations by cytogenetic analysis as previously described.^5^

**SRS_I_ and BALL score**

The SRS_I_ score was calculated giving points to β2-M (1 point for cases with β2-M >5 mg/L), hemoglobin (2 point for men with hemoglobin <11 g/L and 2 point for women with hemoglobin <12 g/L), LDH (2 point for cases with LDH >upper limit of normal); patients were grouped into low-risk (score 0), intermediate-risk (score 1-3), high-risk (score 4-5) (Supplementary Table 1), as previously reported.^6^

The BALL score was calculated giving points to β2-M (1 point for cases with β2-M >5 mg/L), hemoglobin (1 point for men with hemoglobin <11 g/L and 1 point for women with hemoglobin <12 g/L), LDH (1 point for cases with LDH > upper limit of normal) time from last therapy (1 point for cases receiving ibrutinib <24 months from last therapy). Patients were grouped into low-risk (score 0-1), intermediate-risk (score 2-3), high-risk (score 4), (Supplementary Table 1).^7^

**Statistical analysis**

For categorical variables, statistical comparisons were performed using two-way tables for the Fisher’s exact test and multi-way tables for the Pearson’s Chi-square test. OS and TTFT analyses were performed using the Kaplan-Meier method. For OS, the time interval was measured from the day of CLL diagnosis until death from all causes or last follow-up. For TTFT from the day of CLL diagnosis until the start of therapy or last follow-up. Statistical significance of associations between individual variables and survival was calculated using the log-rank test. The prognostic impact for the outcome variable was investigated by univariate and multiple Cox regression analysis. Results are expressed as hazard ratios (HR) and 95% confidence intervals (CI). A value of P<0.05 was considered significant. The predictive accuracy of the prognostic models was quantified by calculating the Harrell C-statistic, the explained variation on outcome (an index combining calibration and discrimination).^8^ Data analysis was performed by STATA for Windows v.9 and SPSS Statistics v.21.

**Supplementary References**

1. Langerak AW, et al. Immunoglobulin sequence analysis and prognostication in CLL: guidelines from the ERIC review board for reliable interpretation of problematic cases. Leukemia. 2011;25(6):979-84.
2. Damle RN, et al. Ig V gene mutation status and CD38 expression as novel prognostic indicators in chronic lymphocytic leukemia. Blood. 1999;94(6):1840-7.
3. Hamblin TJ, Davis Z, Gardiner A, Oscier DG, Stevenson FK. Unmutated Ig V(H) genes are associated with a more aggressive form of chronic lymphocytic leukemia. Blood. 1999;94(6):1848-54.
4. Döhner H, et al. Genomic aberrations and survival in chronic lymphocytic leukemia. N Engl J Med. 2000;343(26):1910-6.
5. Rigolin GM,  et al. Chromosome aberrations detected by conventional karyotyping using novel mitogens in chronic lymphocytic leukemia with “normal” FISH: correlations with clinicobiologic parameters. Blood. 2012;119(10):2310-13.
6. Gentile M, et al. Survival-risk score for real-life relapsed/refractory chronic lymphocytic leukemia patients receiving ibrutinib. A Campus CLL study. Leukemia. 2020; doi: 10.1038/s41375-020-0833-x. [Epub ahead of print].
7. Soumerai JD, et al. [Prognostic risk score for patients with relapsed or refractory chronic lymphocytic leukaemia treated with targeted therapies or chemoimmunotherapy: a retrospective, pooled cohort study with external validations.](https://www.ncbi.nlm.nih.gov/pubmed/31109827) Lancet Haematol. 2019;6(7):e366-e374.
8. Tripepi G, et al. Risk prediction models. Nephrol Dial Transplant*.* 2013;28:1975-80.

**Supplementary Table 1. SRS_I_ and BALL score**

| **SRS_I_** | | | |  |
| --- | --- | --- | --- | --- |
|  | 0 points | 1 point | | 2 points |
| β2-M, mg/L | ≤5 | >5 | |  |
| Hemoglobin | >11 g/L for women  >12 g/L for men | - | | ≤11 g/L for women  ≤12 g/L for men |
| LDH | ≤UNL | - | | >UNL |
| Total score 0= low-risk; score 1-3= intermediate-risk; score 4-5= high-risk | | | | |
|  | | | | |
| **BALL score** | | | | |
|  | 0 points | | 1 point | |
| β2-M, mg/L | ≤5 | | >5 | |
| Hemoglobin | >11 g/L for women  >12 g/L for men | | ≤11 g/L for women  ≤12 g/L for men | |
| LDH | ≤UNL | | >UNL | |
| Time from last therapy | ≥24 months | | <24 months | |
| Total score 0-1= low-risk; score 2-3= intermediate-risk; score 4= high-risk. | | | | |

Abbreviations: β2-M: β-2 microglobulin; ULN: upper limit of normal.

**Supplementary Table 2. Patients’ clinical features**

| Features | Idela-R  (N=142) |
| --- | --- |
|  | **N (%)** |
| Age, years |  |
| ≤65 | 31 (21.8) |
| >65 | 111 (78.2) |
| Sex |  |
| Male | 98 (69) |
| Female | 44 (31) |
| No of previous therapies |  |
| 1 | 29 (20.4) |
| 2-3 | 71 (50) |
| >3 | 42 (29.6) |
| Binet stage |  |
| A | 8 (5.6) |
| B | 57 (40.1) |
| C | 77 (54.3) |
| Hemoglobin g/L |  |
| >120 for men | 49 (50) |
| <120 for men | 49 (50) |
| >110 for women | 25 (56.8) |
| <110 for women | 19 (43.2) |
| β2-M (mg/L) |  |
| ≤3.5 | 55 (38.7) |
| >3.5 | 87 (61.3) |
| <5 | 83 (58.5) |
| ≥5 | 59 (41.5) |
| LDH |  |
| Normal | 88 (62) |
| Elevated | 54 (38) |
| *IGHV* mutational status |  |
| Mutated | 59 (41.6) |
| Unmutated | 83 (58.4) |
| Time from last therapy |  |
| >24 months | 33 (23.2) |
| <24 months | 109 (76.8) |
| 17p deletion |  |
| No | 104 (73.2) |
| Yes | 38 (26.8) |
| Complex karyotype |  |
| Negative | 45 (31.7) |
| Positive | 15 (10.6) |
| Not evaluated | 82 (57.3) |
| SRS_I_ score |  |
| Low | 36 (25.4) |
| Intermediate | 76 (53.5) |
| High | 30 (21.1) |
| BALL score |  |
| Low | 46 (32.4) |
| Intermediate | 77 (54.2) |
| High | 19 (13.4) |

**Supplementary Figure legend**

**Supplementary Figure 1.** Overall survival of the entire population of 142 CLL patients according to BALL score.
