# Supplementary figures and images for: Validation of a survival-risk score (SRS) in relapsed/refractory CLL patients treated with idelalisib–rituximab
Source: Blood Cancer J. 2020 Sep 16;10(9):92. doi: 10.1038/s41408-020-00358-3 (PMC7494850; doi:10.1038/s41408-020-00358-3)

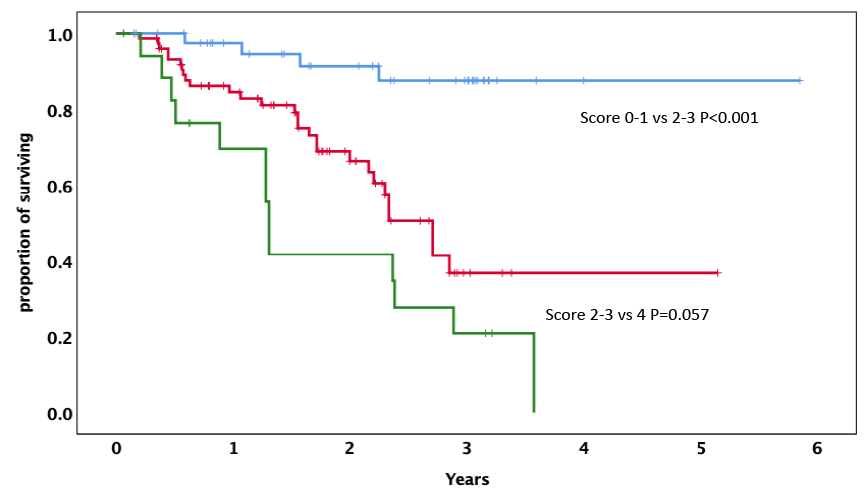

Supplement: Supplementary file 2 — Supplementary Figure 1 [file 41408_2020_358_MOESM2_ESM.tif]
